# Expertise and the Interaction between Different Perceptual-Cognitive Skills: Implications for Testing and Training

**DOI:** 10.3389/fpsyg.2016.00792

**Published:** 2016-05-25

**Authors:** André Roca, A. Mark Williams

**Affiliations:** ^1^Expert Performance and Skill Acquisition Research Group, School of Sport, Health and Applied Science, St. Mary's UniversityTwickenham, UK; ^2^Centre for Cognitive Neuroscience, College of Health and Life Sciences, Brunel University LondonLondon, UK

**Keywords:** expert performance, skill acquisition, representative task design, anticipation, decision making

In recent years, there has been considerable interest in exploring the nature of perceptual-cognitive expertise across a range of domains, such as emergency medicine (e.g., McRobert et al., 2013), music (e.g., Pearce, 2015), military combat (e.g., Williams et al., 2008), aviation (e.g., Palmisano and Gillam, 2005), and sport (e.g., Williams and Ford, 2008; Roca et al., 2011). *Perceptual-cognitive expertise* refers to the ability of an individual to identify and process environmental information for integration with existing and ongoing knowledge to facilitate response selection (Marteniuk, 1976). For example, in sport, experts have been shown to possess superior perceptual-cognitive skills when compared with their less-expert counterparts. These skills include: (a) *postural cue usage*, which is the ability to pick-up early or advance cues emanating from the postural orientation of opponents (Savelsbergh et al., 2005; Abernethy and Zawi, 2007); (b) *pattern recognition*, which is the capacity to recognize task-specific patterns and structure in an evolving situation (Williams et al., 2006; North et al., 2009); and (c) *situational probabilities*, which is the superior ability to generate more accurate predictions as to what others are likely to do in any given situation (Farrow and Reid, 2012). While considerable effort has been devoted to identifying each of these perceptual-cognitive skills using controlled, experimental tasks, limited effort has been devoted to exploring the complex interactions between these skills and the implications for testing and training across domains.

## The interaction between different perceptual-cognitive skills: implications for performance testing

The majority of researchers attempting to examine the importance of perceptual-cognitive expertise have done so using simplistic and contrived paradigms that isolate single perceptual and/or cognitive skills from the others (for a review, see Williams et al., 2011). The design of well controlled and reproducible experiments with good ecological and external validity has always been a challenge for scientists, particularly in the sport domain, given the highly dynamic and rapidly changing nature of the competition setting as well as the emotional and complex perceptuo-motor demands of performance (for a review, see van der Kamp et al., 2008). The removal of key elements and environmental constraints from the stimulus display may force performers to use processes which they do not normally use to solve a task, reducing the possibility of identifying the specific and complex perceptual-cognitive skills employed and how the underlying processes interact and are mediated by expertise (Williams and Ericsson, 2005; Dicks et al., 2009). For example, an investigation by North et al. (2009) into the perceptual-cognitive processes underpinning anticipation and pattern recognition judgments revealed systematic differences in visual behaviors (e.g., number of fixation locations) when participants are required to anticipate what will happen next compared to when they have to recognize sequences of play.

In a follow-up study, North et al. (2011) reported comparable results using retrospective verbal report protocols. When asked to anticipate, rather than recall task-specific situations, participants verbalized more complex stimuli, actions, and cognitions, suggesting that some different mediating processes underpin these two types of judgments. Similarly, Gorman et al. (2015) examined the relationship between pattern recall and decision making in a dynamic and complex team-sport environment by analyzing gaze strategies. The visual search data highlighted significant disparities in the processing strategies (i.e., fewer fixations of longer duration during the decision-making compared with the recall task), indicating that recall skill may utilize different underlying perceptual processes compared to those required for superior decision making in the normal competitive environment. The authors indicated that recognition may be only one of a myriad of perceptual-cognitive skills that contribute to the expert's ability to select more accurate responses within the real performance setting.

A degree of caution is required when employing a task paradigm that does not directly involve the same processes that are normally used during actual performance. When playing in a competitive soccer match, for example, players are required to anticipate and make appropriate decisions but are not required inevitably to recognize or recall sequences of play. This latter issue has direct implications for the methods employed to capture performance. While traditionally more reductionist approaches have been crucial in helping to identify some of the key underlying perceptual-cognitive skills and mechanisms, including the ability to answer more empirical and theoretical questions, there remains a need to look at how these skills interact during performance. In order to explore this later issue, we may need to use more realistic and ecologically valid designs/paradigms involving multidimensional process-tracing measures such as eye movement recording, verbal protocol analysis, and representative environmental and/or task constraint manipulations.

**Figure 1:**
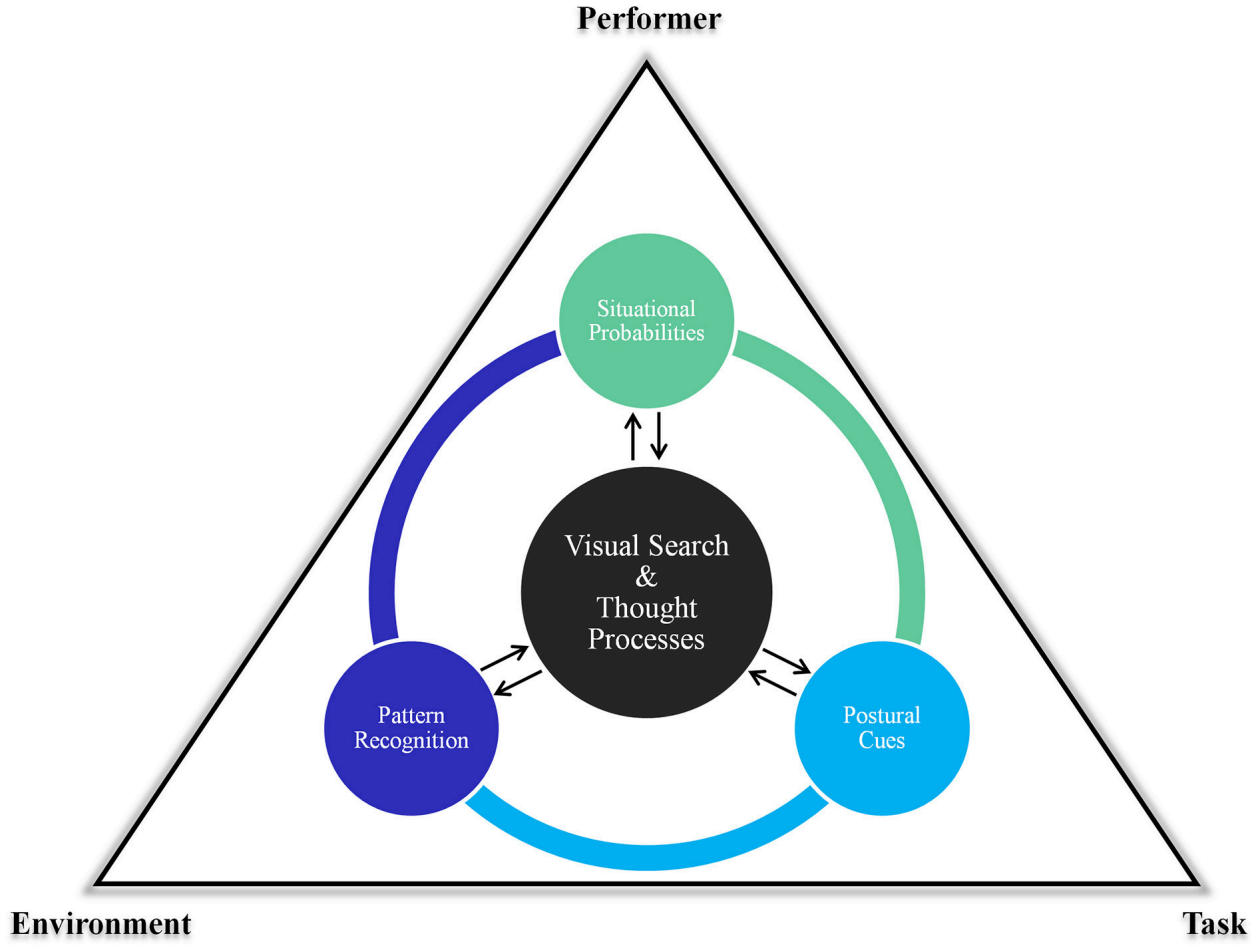
**The interactive relationship between the different perceptual-cognitive skills and processes under various constraints during anticipation and decision making (adapted from Williams, 2009)**.

Williams (2009) made a first attempt to present a framework to illustrate how the different perceptual-cognitive skills may interact in a continuous, dynamic, and reciprocal manner during performance. This framework is based on the notion that the relative importance of these perceptual-cognitive skills may vary based on a range of constraints related to the task, situation, and performer; each being underpinned by different visual search behaviors and thought processes (e.g., see Figure 1). In certain scenarios, it is plausible that performers may rely solely on the ability to process information arising from an opponent's postural orientation (e.g., a goalkeeper facing a particular penalty taker for the very first time), yet under more dynamic and severe time pressure situations (e.g., open play) it is likely that different perceptual and cognitive skills interact with each other in a changing and evolving fashion to facilitate appropriate anticipation and decision making.

There have been attempts to systematically examine how the relative importance of each of these skills varies across different domain-specific tasks and situations. Roca et al. (2013) used a novel approach to examine how perceptual-cognitive skills such as postural cues, pattern recognition, and situational probabilities interact during performance in a representative and dynamic anticipation and decision-making task. Skilled and less-skilled players interacted with 11 vs. 11 soccer sequences filmed from the perspective of a central defender under two different task constraints in which the ball was either located in the offensive or defensive half of the pitch (*far* vs. *near* conditions). Participants' eye movements and retrospective verbal reports of thinking were recorded across these conditions. Verbal report data highlighted a continuous and highly-dynamic interaction between the different perceptual-cognitive skills during performance, with their relative importance varying as a function of the unique constraints of the task environment. In the far task, skilled participants made more statements that referred to the relational information between players (i.e., pattern recognition), whereas in the near task they verbalized more thought processes that related to the postural orientation of opponents or teammates, as well as to what their opponents were likely to do in advance of the actual event (i.e., situational probabilities). These data generally supported the conclusions drawn from the eye-movement results. These findings imply that anticipation and decision making are dependent on a range of perceptual-cognitive skills, which vary in importance from one situation to the next.

A proposal is that several additional factors and constraints like emotions and specific context influence how important different perceptual-cognitive skills are at any given moment when anticipating or making decisions. Cocks et al. (2015) examined the involvement of high- (e.g., situational probabilities) and low-level cognitive processes (e.g., postural cue usage) during a dynamic, time-constrained tennis anticipation task and how their importance may interact with anxiety. Anxiety had an impact on the ability of skilled performers to use situation-specific probabilistic information, suggesting that anxiety may have impacted more greatly on the processing of high-level cognitive processes rather than the pick-up of low-level biological motion information from an opponent during anticipation.

Furthermore, it is important to highlight that the use of different perceptual-cognitive skills are likely to be coupled to the action capabilities of each individual (i.e., physical and functional constraints). Researchers have revealed that differences in action capabilities (e.g., faster vs. slower goalkeepers) affect the timing and accuracy of performers' movement response behaviors, thus constraining the coupling of movements to different informational sources/cues (see Dicks et al., 2010).

## The interaction between different perceptual-cognitive skills: implications for training

A recent call has been made to highlight the need for researchers to work toward a better understanding of how different sources of contextual information interact and influence anticipatory or decision-making behavior (see Cañal-Bruland and Mann, 2015). An interesting question would be to know when and how different sources of information such as postural (kinematic) cues and situational probability (contextual) impact on anticipation (e.g., see Triolet et al., 2013; Murphy et al., 2015) and decision making. Triolet et al. (2013) examined the nature and frequency of anticipation and how spatio-temporal constraints impact on these behaviors in professional tennis using descriptive data. They proposed that very early anticipation behaviors occur when players are able to use significant context-specific information before the opponent's stroke. On the other hand, when such information is not available, anticipation happens closer to the moment of ball-racket contact suggesting that the information used is more likely to be based on the opponents' postural orientation in preparation of the stroke.

Additionally, the significance of one perceptual-cognitive skill over another may be affected by the score and time left in a game (e.g., see Farrow and Reid, 2012) as well as the continual access to other contextual sources such as an opponent passing pattern or move/shot probability. Such knowledge is of vital importance to assist in the design and implementation of effective and systematic training interventions to facilitate the acquisition of perceptual-cognitive expertise. If we can increase understanding of how and when the relative importance of different perceptual-cognitive skills become more or less significant it will be possible to design more relevant and information-specific training programs. For example, if we know when pattern recognition skill becomes more influential in a particular moment or context [e.g., ball being located in the opposition half of the pitch (i.e., far situation; see Roca et al., 2013)], then we could train individuals by primarily highlighting the critical environmental cues that can facilitate the recognition of pattern for that particular performance context without the need to manufacture the task paradigm.

A large portion of training interventions have attempted to improve a very specific perceptual-cognitive component or skill (e.g., postural cues usage, pattern recognition, or situational probabilities; for a review, see Causer et al., 2012) by generalizing it across different tasks, situations or context within the performance environment. It is plausible that such training programs may have overemphasized the relative value of some of these skills to actual performance within more specific and unique environmental constraints. An important principle that should be kept in mind when attempting to design perceptual-cognitive skill training interventions is the specificity of practice and, in particular, the notion of transfer-appropriate processing (Schmidt and Lee, 2011). The idea is that conditions in practice are said to be effective to the extent that they engage processing demands (i.e., visual search, recognition, decision making, movement action) for the performer that are the same as required in the transfer environment (i.e., competition).

## Conclusion

We briefly outlined some recent evidence for the existence of a highly-dynamic and continuous interaction between different perceptual-cognitive skills during performance, with their relative importance varying as a function of various constraints to facilitate anticipation and decision making. These skills include the ability to pick up early or advance cues emanating from the postural movements of opponents, to identify task-specific structure or patterns within an evolving situation, and to generate accurate “a priori” expectations of events likely to unfold. Findings have implications for the manner in which scientists and practitioners try to capture, examine, and/or enhance perceptual-cognitive expertise across various sporting and non-sporting domains. In future, greater efforts should be made to examine the more complex nature of perceptual-cognitive expertise by exploring how each of the different perceptual-cognitive skills interact and how other factors like context, task, and emotions influence these interactions. We consequently would like to encourage more people to try and develop methods that better reflect the multifaceted and complex nature of anticipation and decision making.

## Author contributions

AR wrote the initial draft of the manuscript; AR and AMW revised the manuscript together; both authors gave final approval for publication.

### Conflict of interest statement

The authors declare that the research was conducted in the absence of any commercial or financial relationships that could be construed as a potential conflict of interest.
